# Clinical features and misdiagnosis analysis of pediatric inguinal direct hernia: a single-center retrospective study of 23 cases

**DOI:** 10.3389/fped.2026.1837157

**Published:** 2026-07-07

**Authors:** Jun Shu, Jung Yang, Hongqiang Bian, Fei Peng, Kai Zheng, Haibin Wang, Hongxi Guo, Huan Li, Haiyan Lei

**Affiliations:** Gastrointestinal Surgery, Wuhan Children's Hospital (Wuhan Maternal and Child Healthcare Hospital), Tongji Medical College, Huazhong University of Science & Technology, Wuhan, China

**Keywords:** children, direct hernia, inguinal hernia, laparoscopy, reoperation

## Abstract

**Objective:**

To investigate the clinical characteristics and common etiologies of misdiagnosis in pediatric direct inguinal hernia.

**Methods:**

A retrospective analysis was conducted on 23 pediatric cases of direct inguinal hernia treated at Wuhan Children's Hospital, affiliated with Tongji Medical College of Huazhong University of Science and Technology, from September 2020 to September 2025. The cohort comprised 18 male and 5 female patients, aged from 3 months to 13 years and 6 months, with a median age of 4 years and 5 months.

**Results:**

All 23 cases were initially misdiagnosed as indirect inguinal hernia preoperatively. Among these, 10 cases were correctly identified as direct inguinal hernia during the initial surgical intervention. Eleven patients underwent high ligation of the hernia sac due to misdiagnosis, subsequently developing ipsilateral inguinal masses postoperatively, which were confirmed as direct inguinal hernia during laparoscopic reoperation after an average interval of 4.7 months. Two patients were found to have contralateral direct inguinal hernia incidentally during their second surgery for ipsilateral indirect inguinal hernia, with simultaneous repair performed. Surgical management included laparoscopic ligation of the direct hernia orifice combined with medial umbilical ligament reinforcement for direct hernias, and laparoscopic high ligation of the hernia sac for indirect hernias. A total of 26 direct hernia repairs were performed in 23 patients, including 12 left-sided, 14 right-sided, 3 bilateral, and 3 Pantaloon hernias. Postoperative follow-up ranged from 1 month to 5 years, with no instances of recurrence or complications such as hematoma, wound infection, or testicular atrophy.

**Conclusion:**

The diagnosis of direct inguinal hernia presents significant challenges, with intraoperative misdiagnosis being the primary cause of reoperation in pediatric cases. Intraoperative downward and inward traction of the medial umbilical ligament facilitates better visualization of the direct hernia ring. Laparoscopic management of pediatric direct inguinal hernia demonstrates safety, efficacy, and low complication rates, warranting broader clinical application.

## 1 Introduction

Inguinal hernia represents the most prevalent surgical condition in pediatric populations, with indirect inguinal hernias constituting nearly all cases, attributable to congenital patent processus vaginalis, accounting for over 95% of instances (1). The incidence of direct inguinal hernias is remarkably low, traditionally estimated at approximately 1% of pediatric hernias, with up to 60% of these cases being diagnosed intraoperatively (2). Direct and indirect hernias exhibit highly similar clinical presentations, leading to a substantial misdiagnosis rate. This often results in postoperative recurrence of inguinal masses in children, necessitating multiple surgical interventions and causing significant distress to both the patients and their families. This study conducted a retrospective analysis of clinical data from 23 pediatric cases of direct inguinal hernia treated at Wuhan Children's Hospital, affiliated with Tongji Medical College of Huazhong University of Science and Technology, from September 2020 to September 2025. The aim was to investigate and disseminate insights regarding the clinical characteristics and common etiological factors contributing to misdiagnosis in pediatric direct inguinal hernia.

## 2 Materials and methods

### 2.1 General information

A cohort of 23 pediatric patients diagnosed with direct inguinal hernia was admitted to our institution between September 2020 and September 2025. The patient population comprised 18 male individuals (78.3%) and 5 female individuals (21.7%), with an age range spanning from 3 months to 13 years and 6 months, and a median age of 4 years and 5 months.

### 2.2 Diagnostic methods

Currently, the definitive diagnosis of direct inguinal hernia predominantly relies on laparoscopic surgical exploration. The diagnostic process initiates with comprehensive medical history collection, including characteristic symptoms such as recurrent inguinal masses that exacerbate during episodes of crying. Physical examination typically reveals masses localized in the medial inguinal region, specifically within Hesselbach's triangle, which demonstrate limited spontaneous reducibility. Auxiliary diagnostic modalities include ultrasonographic examination, which facilitates the observation of hernia contents and the localization of the hernia ring, thereby assisting in the preliminary differentiation between direct and indirect hernias. However, the diagnostic sensitivity of ultrasound is constrained and significantly influenced by the operator's technical expertise. Intraoperative diagnosis remains pivotal in confirming direct inguinal hernia. Laparoscopic exploration provides definitive visualization of the hernia ring's anatomical position within Hesselbach's triangle, medial to the inferior epigastric vessels, thereby distinguishing it from the characteristic lateral position observed in indirect hernias.

### 2.3 Surgical methods

**Figure 1:**
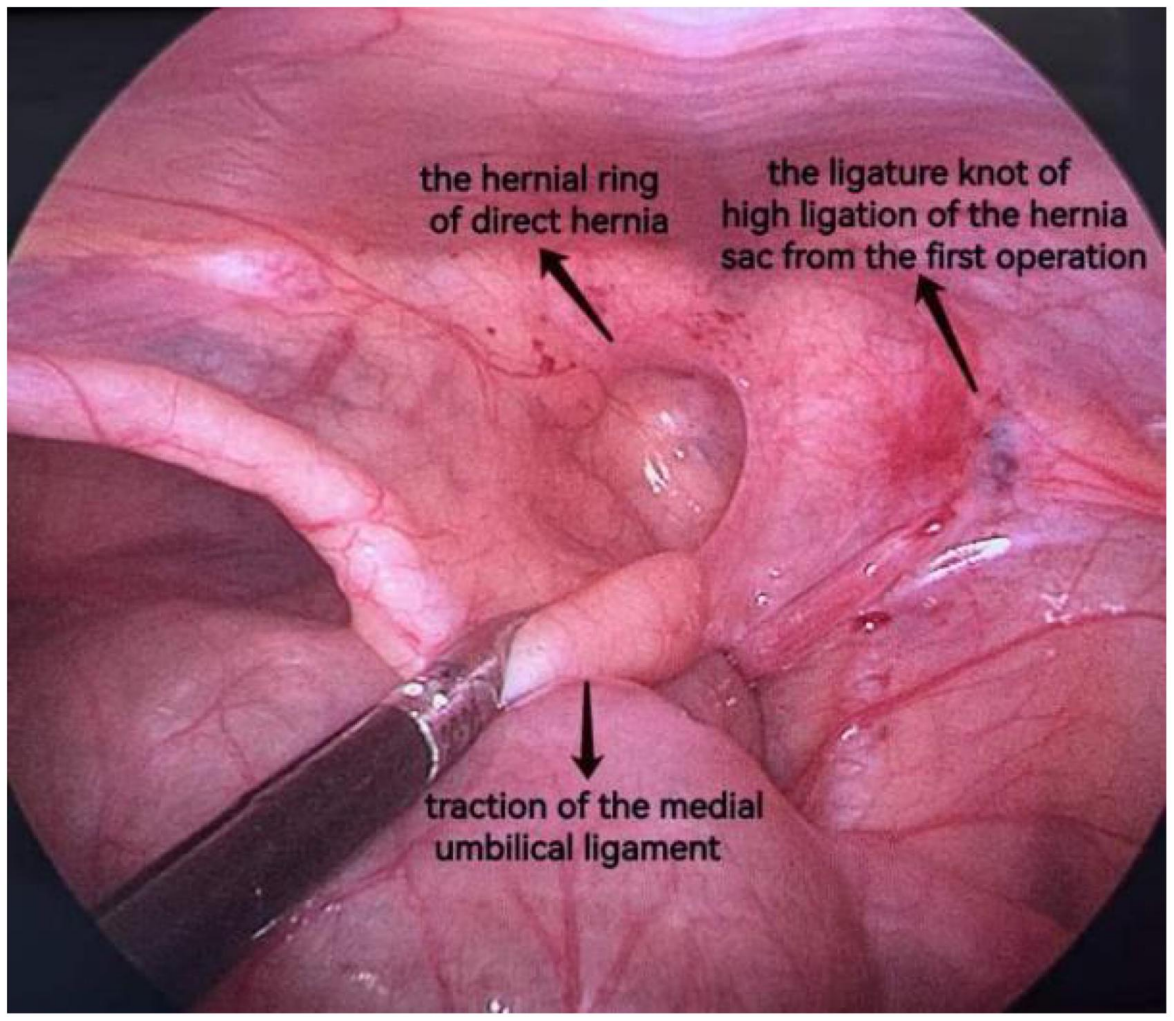
Illustrates a npediatric patient undergoing secondary surgical intervention (case 13 in Table 2). Intraoperative traction of the medial umbilical ligament facilitates optimal exposure of the hernial ring in direct hernia cases, while the ligature knot from the primary high ligation of the hernia sac is clearly visible.

**Figure 2:**
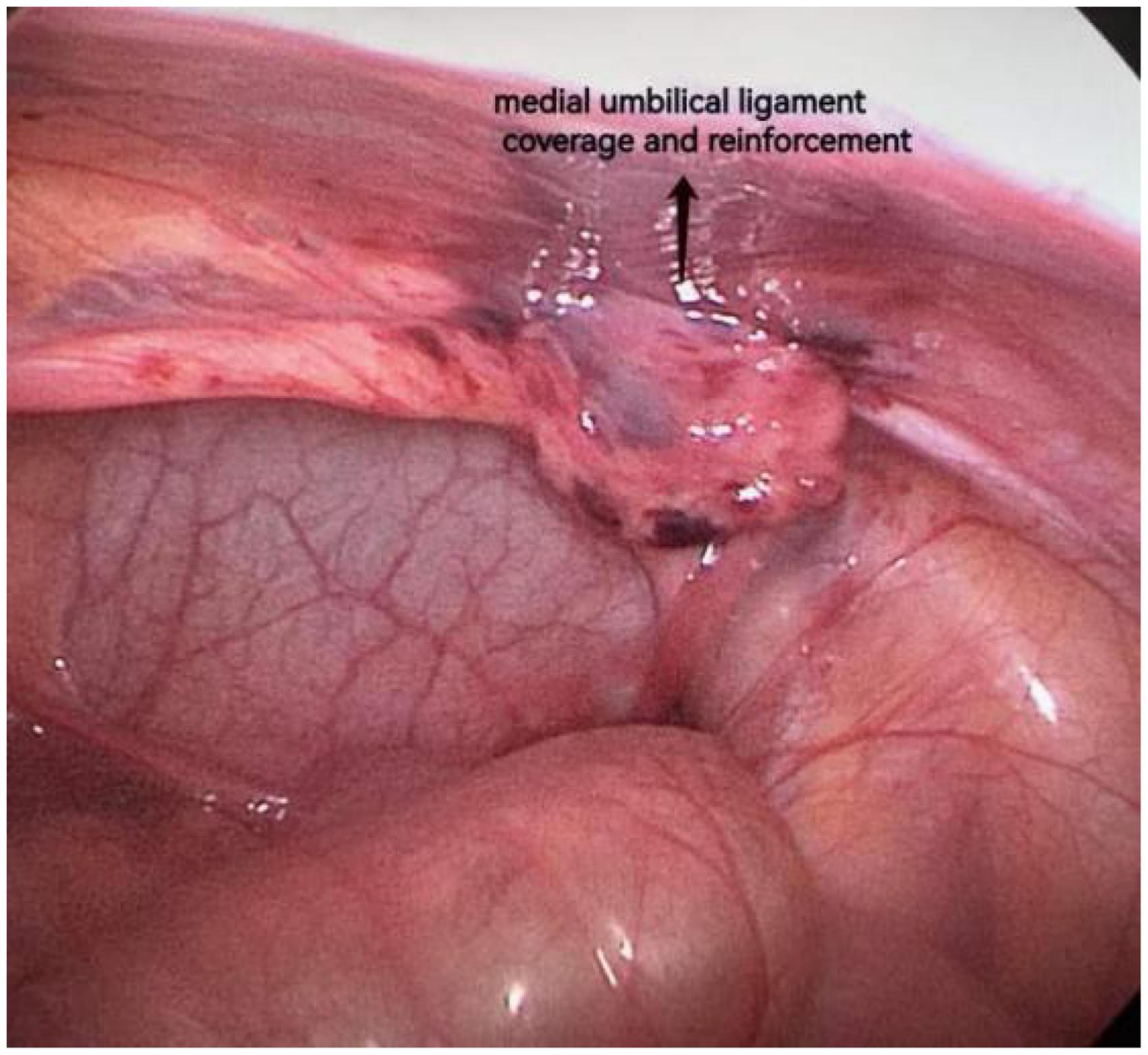
Depicts a pediatric patient undergoing reoperation (case 13 in Table 2), demonstrating the surgical procedure following ligation of the direct hernia ring, which is subsequently covered and reinforced using the medial umbilical ligament.

**Figure 3:**
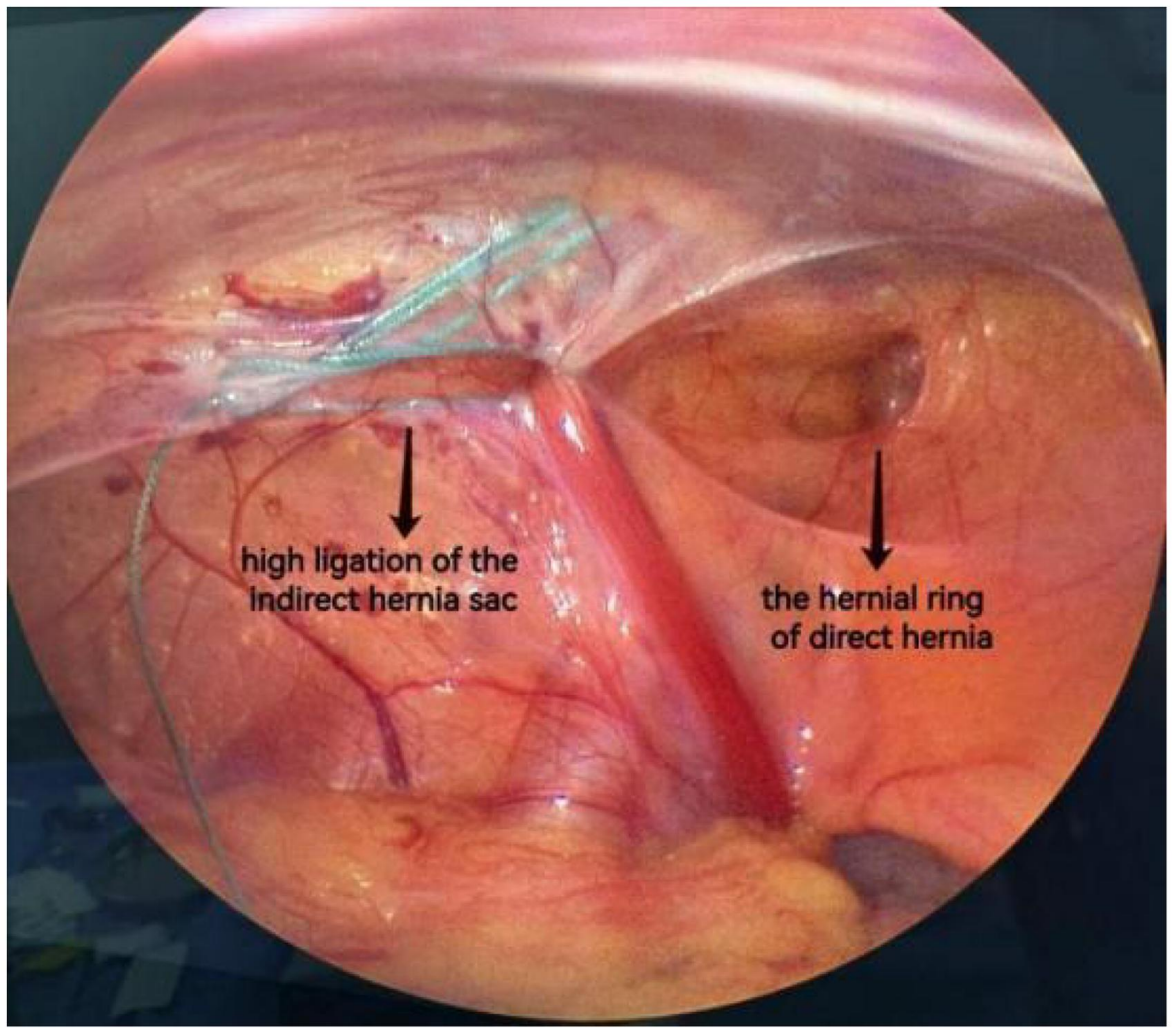
Presents a pediatric case of pantaloon hernia (case 10 in Table 1), where high ligation of the indirect hernia sac was performed, revealing a direct hernia ring orifice on its medial aspect.

All pediatric patients with intraoperatively confirmed direct inguinal hernia underwent laparoscopic direct hernia orifice ligation combined with medial umbilical ligament coverage and reinforcement repair. The surgical procedure was conducted as follows: The patient was positioned in a supine orientation with a 15°Trendelenburg tilt under endotracheal general anesthesia. Surgical access was established through bilateral 3 mm arc-shaped incisions along the umbilical ring. A Veress needle was utilized to establish carbon dioxide pneumoperitoneum, maintaining intra-abdominal pressure between 8 and 12 mmHg. Subsequently, 3.5 mm Trocars were inserted bilaterally, with the laparoscope positioned on the right side and an atraumatic grasper on the left. Laparoscopic exploration confirmed the hernia sac's location medial to the inferior epigastric artery, verifying the diagnosis of direct inguinal hernia. It is crucial to emphasize that certain direct inguinal hernias may present diagnostic challenges intraoperatively. In such instances, downward and inward traction of the medial umbilical ligament is essential to adequately expose the direct hernia orifice (Figure 1), thereby preventing potential missed diagnoses. The surgical technique proceeded as follows: The hernia needle was advanced extraperitoneally along the medial aspect of the hernia orifice, completing a semicircular trajectory before entering the abdominal cavity, leaving one end of the silk suture intra-abdominally. The needle was then reintroduced, traversing the inferior epigastric artery and completing a semicircular path along the lateral aspect of the hernia orifice before penetrating the peritoneum, thereby retrieving the first silk suture for extracorporeal knotting. Subsequent steps involved reintroduction of the hernia needle carrying the silk suture from the lateral aspect of the hernia orifice, piercing the medial umbilical ligament to secure the suture in position. The hernia needle was then withdrawn to the extraperitoneal space and re-entered the abdominal cavity 1 cm distal to the initial puncture site, facilitating suture retrieval and completion of the coverage procedure (Figure 2). For patients presenting with concurrent indirect inguinal hernia, laparoscopic high ligation of the hernia sac was performed concomitantly during the procedure (Figure 3).

**Table 1:** Clinical data of 10 children with direct hernia who underwent one surgery.

Case	Gender	Age	Preoperative diagnosis	Intraoperative diagnosis	Surgical procedure
1	Female	7 years 5 months	Left indirect hernia	Right indirect hernia Left direct hernia	Laparoscopic high ligation of the right hernial sac + laparoscopic ligation of the left hernial ring+reinforcement of medial umbilical ligament
2	Female	5 years 5 months	Left hydrocele	Left direct hernia	Laparoscopic ligation of left hernia ring + reinforcement of medial umbilical ligament
3	Male	3 years 10 months	Left indirect hernia	Left direct hernia	Laparoscopic ligation of left hernia ring + reinforcement of medial umbilical ligament
4	Male	13 years 6 months	Left hydrocele	Left hydrocele Right direct hernia	Laparoscopic high ligation of the left processus vaginalis + laparoscopic ligation of the right hernial ring + reinforcement of medial umbilical ligament
5	Male	3 years 7 months	Right indirect hernia	Bilateral direct hernia	Laparoscopic bilateral ligation of the hernia ring + reinforcement of medial umbilical ligament
6	Male	3 months	Left indirect hernia	Left direct hernia	Laparoscopic left ligation of the hernia ring + reinforcement of medial umbilical ligament
7	Male	6 years 4 months	Right indirect hernia	Right indirect hernia Right direct hernia (Pantaloon hernia)	Laparoscopic high ligation of the right hernial sac + laparoscopic ligation of the right hernial ring + reinforcement of medial umbilical ligament
8	Male	11 years 11 months	Right indirect hernia	Right direct hernia	Laparoscopic right ligation of the hernia ring + reinforcement of medial umbilical ligament
9	Male	9 months	Bilateral indirect hernia	Bilateral indirect hernia Right direct hernia (Pantaloon hernia)	Laparoscopic bilateral high ligation of the hernial sacs + laparoscopic ligation of the right hernia ring + reinforcement of medial umbilical ligament
10	Female	8 years	Left indirect hernia	Bilateral indirect hernia Left direct hernia (Pantaloon hernia)	Laparoscopic bilateral high ligation of the hernial sacs + laparoscopic ligation of the left hernial ring + reinforcement of medial umbilical ligament

**Table 2:** Clinical data of 13 children with direct hernia who underwent two surgeries.

Case	Gender	Age	Preoperative diagnosis of 1st surgery	Intraoperative diagnosis of 1st surgery	Interval time of the 2 surgeries	Preoperative diagnosis of 2nd surgery	Intraoperative diagnosis of 2nd surgery
1	Male	4 years 1 month	Right indirect hernia	Right indirect hernia	1 year	Right recurrent indirect hernia	Right direct hernia
2	Male	8 years 6 months	Left indirect hernia	Left indirect hernia	1 month	Left recurrent indirect hernia	Left direct hernia
3	Male	4 years 8 months	Right indirect hernia	Bilateral indirect hernia	4 months	Right recurrent indirect hernia	Right direct hernia
4	Male	8 years	Right hydrocele	Bilateral hydrocele	2 weeks	Right recurrent hydrocele	Right direct hernia
5	Male	3 years 3 months	Bilateral indirect hernia	Bilateral indirect hernia	1 month	Bilateral recurrent indirect hernia	Bilateral direct hernia
6	Female	1 year 6 months	Left indirect hernia	Left indirect hernia	1 year	Left recurrent indirect hernia	Left direct hernia
7	Male	9 years 4 months	Bilateral indirect hernia	Bilateral indirect hernia	1 month	Bilateral recurrent indirect hernia	Bilateral direct hernia
8	Male	4 years 5 months	Right indirect hernia	Bilateral indirect hernia	1 year	Right recurrent indirect hernia	Right direct hernia
9	Male	2 years 3 months	Right indirect hernia	Right indirect hernia	5 months	Right recurrent indirect hernia	Right recurrent indirect hernia, left direct hernia
10	Male	2 years 1 month	Right indirect hernia	Bilateral indirect hernia	6 months	Right recurrent indirect hernia	Right direct hernia
11	Male	13 years	Left indirect hernia	Left indirect hernia	11 years	Right indirect hernia	Right indirect hernia, left direct hernia
12	Male	3 years 4 months	Bilateral indirect hernia	Bilateral indirect hernia	2 months	Right recurrent indirect hernia	Left recurrent indirect hernia, right direct hernia
13	Female	2 years 6 months	Right indirect hernia	Bilateral indirect hernia	1 week	Right recurrent indirect hernia	Right direct hernia

## 3 Results

This study enrolled 23 pediatric patients with direct inguinal hernia, all of whom were preoperatively misdiagnosed with indirect inguinal hernia. Initial surgical intervention revealed direct inguinal hernia in 10 cases (Table 1), prompting laparoscopic direct hernia ring ligation with medial umbilical ligament reinforcement. Postoperative analysis identified 11 cases (47.8% recurrence rate, Table 2) of relapse due to initial misdiagnosis. These patients, who had undergone high ligation of the hernia sac under the misdiagnosis of indirect inguinal hernia, presented with ipsilateral inguinal masses and were subsequently confirmed to have direct inguinal hernia through laparoscopic evaluation during reoperation. These cases received laparoscopic direct hernia ring ligation with medial umbilical ligament coverage reinforcement. The interoperative interval ranged from 1 week to 4.7 months (mean: 4.7 months). Two additional cases (Cases 9 and 11, Table 2) underwent initial surgery for indirect inguinal hernia, with contralateral direct inguinal hernia incidentally discovered during subsequent ipsilateral indirect hernia repair. These cases received simultaneous direct hernia repair and were excluded from recurrence statistics. The surgical cohort comprised 26 direct hernia repairs in 23 patients, including 12 left-sided, 14 right-sided, 3 bilateral, and 3 Pantaloon hernias (Cases 7, 9, and 10, Table 1). Surgical parameters included a mean operative time of 28 min, intraoperative blood loss <3 mL, and no iatrogenic complications. Postoperative recovery was uneventful, with oral intake resumed at 2 h and discharge within 24 h. Follow-up ranged from 1 month to 5 years, demonstrating no recurrence or complications such as hematoma, wound infection, or testicular atrophy.

## 4 Conclusion

Clinical direct inguinal hernia represents a rare condition in the pediatric population, constituting 0.2%–3.9% of all inguinal hernia cases. Epidemiological data demonstrate a male predominance, with peak incidence occurring between 6 months and 10 years of age, and a higher prevalence of right-sided compared to left-sided presentations (1, 3–5). Our institutional data from September 2020 to September 2025 revealed 23 cases of direct hernia among 15,730 inguinal hernia surgeries, yielding an incidence rate of 0.15%, which is marginally lower than previously documented rates. The observed gender distribution, age of onset, and laterality patterns in our cohort align with existing literature reports.

Hesselbach's triangle, delineated by the inferior epigastric artery, the lateral border of the rectus abdominis muscle, and the inguinal ligament, is characterized by incomplete and weak muscular coverage. This anatomical feature predisposes to anterior protrusion of abdominal organs through this region, resulting in the formation of direct inguinal hernias. Wright (6) defined five types of direct inguinal hernia: (1) diffuse weakness of the posterior inguinal wall with attenuated transversalis fascia and no significant direct sac; (2) direct and indirect sacs (pantaloon hernia), three cases of this type were documented in this study; (3) a distinct direct sac without an indirect sac, with 20 cases identified in the present study; (4) sliding direct hernia; and (5) generalized destruction of the integrity of the posterior wall of the inguinal canal by a massive indirect hernia widely stretching the internal ring.

The diagnosis of direct inguinal hernia in pediatric patients presents significant challenges, with a notably high rate of misdiagnosis. In the present study, all 23 pediatric cases were initially misdiagnosed as indirect inguinal hernias prior to surgical intervention, resulting in a preoperative misdiagnosis rate of 100%. Among these cases, 10 patients were correctly identified as having direct hernias during the initial surgical procedure. However, 11 patients remained misdiagnosed as indirect hernias during the first surgery and consequently underwent only laparoscopic high ligation of the hernia sac, which led to a postoperative recurrence rate of 47.8%. These cases were subsequently confirmed as direct hernias during secondary surgical procedures. Additionally, 2 patients were found to have direct hernias contralateral to the indirect hernias during the second surgery. The following physical examination techniques have been identified as valuable for enhancing the preoperative diagnostic accuracy of direct hernias in pediatric patients: (1) Persistent protrusion of a mass medial to the internal ring when applying digital pressure to the internal ring of an indirect hernia; (2) The protrusion site of a direct hernia is typically more medial compared to that of an indirect hernia; (3) The base of a direct hernia is generally wider and rarely extends into the scrotum. Relevant reports (7) indicate that during preoperative high-frequency ultrasound examination for adult inguinal hernia, the relationship between the inferior epigastric artery and the hernial sac can be used to differentiate between direct and indirect hernias. However, in pediatric practice, the differentiation between direct and indirect inguinal hernias remains particularly challenging due to the anatomical characteristics of children, including the short inguinal canal (<0.5 cm) and the nearly overlapping internal and external rings (8). Furthermore, the relative rarity of direct hernias in pediatric populations contributes to insufficient clinical awareness among healthcare professionals, including clinicians and sonographers, resulting in frequent diagnostic errors.

The author systematically delineates the underlying factors contributing to diagnostic challenges as follows: (1) The relatively low prevalence and clinical rarity of direct hernias often lead to diagnostic oversight. (2) Anatomical considerations: the partial overlap between the external ring and Hesselbach's triangle creates diagnostic ambiguity between direct and indirect hernias. (3) Insufficient physical examination protocols: not all indirect hernias in male pediatric patients extend into the scrotum; in female pediatric patients, both direct and indirect hernias are confined to the inguinal region. This anatomical similarity often leads clinicians to diagnose inguinal indirect hernia upon palpation of an inguinal mass without further differential diagnosis, constituting a primary factor in preoperative misdiagnosis. (4) While B-mode ultrasonography can provide information regarding hernia contents and hernia ring location, its diagnostic sensitivity is constrained and significantly influenced by operator expertise. Typically, it only confirms the presence of an inguinal hernia without distinguishing between direct and indirect types. In this study, all ultrasonographic examinations identified inguinal hernias but failed to differentiate between direct and indirect variants, representing another significant contributor to preoperative misdiagnosis. (5) Intraoperative cognitive bias: surgeons frequently operate under the assumption of indirect hernias, focusing on identifying the indirect hernia sac and performing high ligation of the processus vaginalis as a symptomatic hernia sac. This approach often lacks thorough exploration of Hesselbach's triangle or involves cursory examination, potentially overlooking the actual hernia sac and leading to early postoperative recurrence. This represents the principal cause of intraoperative misdiagnosis. In the current study, 11 pediatric cases experienced postoperative recurrence due to this factor, necessitating secondary surgical intervention.

Intraoperative laparoscopic exploration constitutes a critical diagnostic procedure for confirming direct inguinal hernia. Laparoscopic visualization enables precise identification of the hernia ring's anatomical position: a medial location relative to the inferior epigastric artery confirms direct inguinal hernia, whereas a lateral position indicates indirect inguinal hernia (9). In laparoscopic management of direct inguinal hernia, preoperative bladder evacuation is routinely implemented due to the anatomical proximity of direct hernia to the bladder compared to indirect hernia; a distended bladder may obscure the internal hernia orifice, potentially leading to intraoperative diagnostic oversight. During surgical intervention, bladder management options include intraoperative catheterization or manual displacement using surgical forceps to facilitate exploration of Hesselbach's triangle. However, our intraoperative observations revealed that even with complete bladder evacuation, direct visualization of the hernia ring remained challenging when the internal ring of indirect hernia appeared closed. Subsequent exploration identified that the median umbilical ligament or medial umbilical ligament frequently obscured the direct hernia ring. In such cases, downward and inward traction of these ligaments proved essential for complete exposure of the hernia ring within Hesselbach's triangle, thereby minimizing diagnostic errors. In our clinical series, these techniques enabled accurate identification in 10 primary surgical cases, obviating the need for secondary procedures, and facilitated successful localization in 13 revision cases. Wafa et al. (10) documented that the direct hernia ring may become obscured following abdominal insufflation, recommending pneumoperitoneum pressure reduction to decrease abdominal wall tension, complemented by external manual compression to enhance hernia ring identification. While this technique was not employed in our study, it represents a valuable methodological consideration.

With the increasing prevalence of minimally invasive hernia repair in pediatric populations (11, 12), laparoscopic identification and repair of direct inguinal hernias has been established as a safe, efficient, and effective therapeutic approach (13). While laparoscopic ligation of the direct hernia ring can temporarily address the peritoneal defect, it fails to correct the underlying abdominal wall defect, potentially leading to increased postoperative recurrence rates (14, 15). Optimal management should incorporate reinforcement and repair of the direct hernia triangle area. In our series, all pediatric patients with direct hernias underwent laparoscopic hernia ring ligation combined with medial umbilical ligament coverage and reinforcement repair, demonstrating several significant advantages: Firstly, the magnified laparoscopic view provides clear visualization of critical anatomical structures including the inferior epigastric vessels, vas deferens, spermatic vessels, and components of the direct hernia triangle, thereby enhancing diagnostic accuracy and minimizing iatrogenic injuries. Secondly, the utilization of the medial umbilical ligament—a naturally occurring robust structure—for coverage and reinforcement of the direct hernia defect eliminates the need for additional dissection and suturing, thereby simplifying surgical procedures, reducing operative time (mean duration: 28 min), and aligning with pediatric tissue characteristics while avoiding potential discomfort associated with traditional tension-based repairs. Finally, the combination of extraperitoneal hernia ring ligation with internal coverage and reinforcement effectively addresses the hernia ring defect while strengthening the weakened abdominal wall area. In our cohort of 26 direct hernia cases, no recurrences were observed during postoperative follow-up ranging from 1 month to 5 years, with no complications such as hematoma, wound infection, or testicular atrophy, confirming the safety, efficacy, and minimally invasive nature of this approach. Compared with previously reported higher recurrence rates in pediatric direct hernias (16), our results demonstrate superior outcomes, potentially attributable to the medial umbilical ligament coverage and reinforcement technique. For cases of Pantaloon hernia, laparoscopy enables simultaneous visualization of both direct and indirect hernia sacs (17), allowing for comprehensive management including hernia ring ligation and reinforcement for direct hernia combined with high ligation of the indirect hernia sac under a single anesthetic episode, thereby avoiding staged procedures and reducing both patient morbidity and healthcare burden.

The incidence of direct inguinal hernia in pediatric populations remains relatively low; however, the clinical significance of secondary surgeries resulting from misdiagnosis warrants considerable attention. This study highlights the technical nuances of intraoperative downward and inward traction of the median or medial umbilical ligaments, a maneuver that not only facilitates hernia ring exposure but also significantly reduces diagnostic inaccuracy, thereby offering valuable clinical guidance. Nevertheless, certain limitations must be acknowledged, including the small sample size (*n* = 23) and the single-center retrospective design, which may introduce selection bias. Future research should focus on expanding the sample size, conducting multicenter prospective studies to validate these findings, and exploring the potential optimization of preoperative imaging techniques, such as dynamic MRI. In summary, laparoscopic technology represents the preferred diagnostic and therapeutic approach for pediatric direct inguinal hernia, demonstrating both diagnostic accuracy and therapeutic reliability, and thus merits broader adoption in pediatric surgical practice.

## Data Availability

The original contributions presented in the study are included in the article/Supplementary Material, further inquiries can be directed to the corresponding author.
